# How to Undertake Research of MHC Utilization in Under-developed Countries? A Case Study of MHC Utilization in Central and Western Rural China

**DOI:** 10.12669/pjms.296.3851

**Published:** 2014

**Authors:** Zhaoxin Wang, Yin Zhang, Minxing Chen

**Affiliations:** 1 Dr. Zhaoxin Wang, PhD, School of Medicine, Tongji University, China.; 2 Dr. Yin Zhang , PhD, Guizhou University of Finance and Economics, China.; 3 Dr. Minxing Chen , PhD, Shanghai Medical Technology Intelligence Institute, China.

**Keywords:** Central and Western Rural China, Maternal Health Care (MHC), Problem, Study Themes, Utilization

## Abstract

***Objective: ***This study was undertaken to address practical problems in maternal health care (MHC) utilization and conduct in-depth study of maternal health services utilization in underdeveloped countries(regions), thus to contribute to the achieving of the UN Millennium Development Goal 5.

***Data Collection:*** After searching and screening based on key words like “MHC” and “utilization”**,** we included 45 English articles and 106 Chinese articles from Pubmed, Medline, China Knowledge Resource Integrated and Wang Fang data base. The research themes, issues, designs, perspectives, dimensions and methods of these dissertations were analyzed.

***Results: ***The development of MHC utilization research can be divided into three phases: Studies of the first phase focused primarily on decreasing MMR, which caused attention to the central and western rural areas maternal health services in China from domestic as well as international community; Studies of the second phase centered around the practical impacts of the implementation of MHC relevant programs and policy, confirming that the implementation of these programs and policies improved MHC service delivery and utilization, and promoted cooperation between researchers and practitioners; Studies of the third phase focused on the quality of MHC service utilization. We also found that the major problem in the current MHC service utilization is the huge gap across regions and the existing researches lack innovation and comparison researches between in different countries.

***Conclusion:*** Research themes of MHC services change regularly. We should grasp the characteristics and defects of current research to increase the innovation of future research and to better response to the problem solving, and thus to provide more valuable reference for the policy and practice of underdeveloped countries and areas.

## BACKGROUND

Improving maternal health is the fifth Millennium Development Goals (MDG 5) passed in United Nation (UN) Millennium Summit in 2000.^1^ Research and practice of improving maternal health in underdeveloped and developing countries including China have always been under the spotlight of international community. Maternal health care (MHC) is a critical measure to reduce maternal mortality rate (MMR), to reduce risk and improve child births quality, and to improve maternal and child quality of life^-^.^2^^-^^5^ Since utilization of MHC services is critical to achieve desirable outcomes and good health^6^, studies focusing on utilization of MHC service have always attracted the attention of both domestic and foreign researchers.

**Fig.1:**
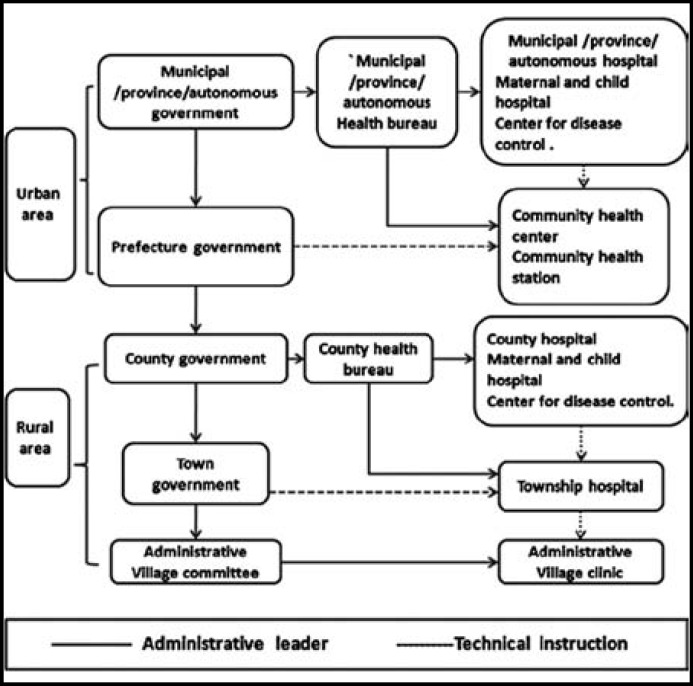
The Administrative Structure and health institutions of China

In 1957, the Chinese Ministry of Health (MOH) established Maternal and Child Health Division, which is the first division specializing in maternal and child health administration, focusing on popularizing new delivery method and protecting women survival; the policies after 1991 mainly focused on reducing MMR. In 2004, the Maternal and Child Health Division changed its name to the Maternal and Child Health and Community Health Division (the name has been in use ever since), primarily responsible for managing, guiding, and supervising maternal and child health according to law, as well as developing and implementing maternal and child health relevant plans.^7^ Maternal and child health service providers adjust according to the establishment of administrative institutions and changes of priorities. So far, China has a stable maternal and child health service delivery networks. The health-care providers are responsible for delivering MHC services in their catchment areas under supervision of government. In urban areas, municipal /province/autonomous hospital, Maternal and child hospital, Center for disease control, community health centers or community health stations are the major MHC providers; in rural areas, County hospital, Maternal and child hospital, Center for disease control, township health centers and village clinics are the major MHC providers. Both of them get administrative and technical instructions from different health institutions, Fig.1.

In China, only a small percentage of rural pregnant women who are in better economic conditions have access to services provided by any level urban medical institutions, which will satisfy this group’s needs in order to generate more income. Most pregnant women choose to use the services provided by local medical institutions. Therefore, medical institutions’ capacity of providing maternal and health service in rural areas exert an influence on the utilization of MHC services. China, as a developing country with a large population, its central and western population is about 786 million (accounting for 58.3% of the nation’s total population); its population of rural areas in central and western China is 458 million (accounting for 60.5% of the central and western areas’ total population).^8^ For a long time, economic and cultural development between urban and rural areas as well as across regions are extremely uneven^9^, with the economic and cultural development level of central and western China, as well as of rural areas, fall behind those of eastern China and urban areas respectively.^10^ MHC service level presents a significant difference in rural and urban areas and across regions. According to certain studies, the service capacities and development level of rural areas in central and western China is roughly the same with those of underdeveloped countries. Thus, there are possibly similar problems in underdeveloped countries with central and western rural areas in China.^11^

This study, taking the MHC research of central and western rural China as an example, combining the practice of MHC supplying and utilization in rural areas, analyzed the research themes, perspectives, target problems, and research methods of previous studies published at home and abroad. We aim to discover defects of current studies, explore strategies of problem solving and the directions of future studies, and thus to provide references for underdeveloped or developing countries.

## METHODS

***MHC: ***According to Chinese Ministry of Health’s defining of *Administration of Maternal Health Care,* MHC refers to medical care service provided for women (preparing for pregnancy or in 42 days within delivery), infants and fetal throughout the process by medical institutions at all levels.^12^ In this study, MHC service utilization refers to women’s utilization of antenatal care (ANC), hospital delivery, and postnatal visits. Prenatal care service includes content, frequency, and timing of relevant health care; hospital delivery service includes understanding of maternal health condition, health guidance, and whole process monitoring of mother and fetal infants; postnatal visits include maternal mental state, physical state and lactation feed, etc.

***Chinese central and western rural areas:*** Chinese central and western areas comprise 12 provinces, autonomous regions, and municipalities, including Western Inner Mongolia and Sichuan province, etc. Central area comprises 8 provinces including Shanxi and Hubei, etc; Rural area comprise three basic administrative agencies—county, township, and village.

***Search strategy and selection criteria: ***The search results in PubMed, Medline, CNKI and Wanfang Data, using key words of “maternal health care”, “prenatal examination”, “hospital delivery” and “postpartum visit”, indicate that it was from 2000 that large numbers of researches began to emerge. Therefore, the literature searching of this study uses 2000 as a starting point. We searched the database of PubMed, Medline, the Social Science Research Network, China Knowledge Resource Integrated Database and Wang Fang data base for articles published from Jan 1, 2000 to Feb 6, 2013. We restricted our search to works published in English or Chinese, and used the search terms “maternal health care”, “antenatal clinic”, “hospital delivery”, “postnatal visits”, “rural areas”, “utilization”, “China”, and combinations of these terms. We collected 146 English articles and 236 Chinese articles in total. Group members’ first round of reading eliminated 85 articles (they either repeated, only having abstract, conference papers, or non-academic). The second round of reading eliminated 146 articles, including 45 ones which were irrelevant with the theme of the research and 101 ones which were case report without specific objects, methods and design. Thus, 45 English articles and 106 Chinese articles were reviewed and analyzed. It was found that the target problems, themes, methods, and design mentioned in these articles were saturated. For example, there were no new research themes or target problems.

## RESULTS

***Development of MHC Utilization theme: ***After summarizing the research theme and target problems of the articles collected, we compared the time of publication, policy involved, and time of program introduction. It was found that the research themes of MHC service utilization from 2000 can be divided into three phases and the target problems and research design under different research themes are different. It must be made clear that it was from the angel of practical problems and researches that we divide the phases and themes, which means that the practical problems of different phases and the response of research to these problems are studied. Therefore, the research themes are not strictly confined to the phases we define.Fig.1.

**Table-I:** Research theme, target problem and research design of different phase

*Phase*	*Research theme*	*Target problem*	*Research design*
The first phase 2000-2003	MHC utilization and MMR decreasing	Relationship between MHC utilization and MMR Analysis of factors influencing MHC utilization	Cross section survey Retrospective analysis Intervention study
The second phase 2004-2010	MHC utilization and MHC program evaluation	Program/policy impact evaluation Program/policy’s influence on MHC utilization	Retrospective analysis Intervention study
The third phase 2011-present	Improving MHC utilization quality	Normalization of MHC utilization Comparative analysis of MHC utilization MHC	Retrospective analysis Cross section survey

MHC: maternal health care,

MMR: maternal mortality rate.

***The first phase (2000-2003) MHC utilization and MMR decreasing: ***From the beginning of 1949 to 2000 or so, instead of using the service provided by medical institutions, most pregnant women in central and western China rural areas chose to give birth at home^13^, which led to high MMR and neonatal mortality rate. Therefore, improving MHC utilization (especially hospital delivery service) and MMR decreasing were the main research theme of this phase. Researchers of this phase carried out large numbers of surveys. A typical example is a case study of a specific area, which carried out a survey of the women and their families and put forward some counter measures basing on the analysis of the relationship between MMR and MHC utilization. Xiaosong Z carried out a survey of maternal mortality of 232 poverty-stricken counties in western China from 1995-2000, which indicated that measures, such as enhancing health education, improving service capacity, promoting MHC utilization, should be taken so as to decrease MMR.^14^ In addition, it had been confirmed by experiments of many counties that delivery assisted by midwife with medical technology and timely emergency obstetric referral could successfully decrease MMR.^15^ Therefore, a number of studied analyzed the relationship between hospital delivery utilization and qualification of hospital staff and institution’ service capacity. For example, Hong X carried out a study analyzing the state of MHC utilization in 46 western poverty-stricken counties. It indicated that township hospitals should strengthen obstetrical department’s service capacity and improve the competence of the staff so as to promote MHC utilization.^16^ It can be said that although studies of this phase did not contribute to MHC utilization in practice and failed to fundamentally promote MHC utilization of central and western rural areas, they did cause the attention of national and international community to MHC utilization in central and western rural areas of China.

***The second phase (2004-2010) MHC utilization and MHC program evaluation: ***In view of the MHC backwardness of the central and western rural areas, international community and Chinese government have increased the investment on maternal and child health. Since the mid 90s, a series of MHC improving programs have been implemented, among which the most influencing ones include “ Reduce MMR and Eliminate Neonatal Tetanus”(Jiang Xiao program for short), “Safe Motherhood” program, “Health VIII” program , SHCRW policy for central and western rural areas. Most of these programs, carried out for certain MHC problems of a specific region and funded, were periodical. Therefore, the implementation of these programs triggered a series of impact and effectiveness evaluation study of MHC utilization.

Liu XN (2010)^17^ use a difference in difference approach assessed the impact of “safe motherhood program” on the MHC utilization of 32 counties in western rural areas, confirming that the program could improve the utilization of ANC. In addition, with the implementation of new rural cooperative medical care system (NRCMS) in 2003, researchers began to concern the impact of NRCMS on MHC utilization. Long Q (2010)^18^ using data (from the statistics centre of the MOH) of 1800 families in 10 provinces of western China, analyzed the influencing factors of MHC utilization before and after the implementation of NRCMS with logistic regression and log-linear model. The research showed that the implementation of NRCMS improved the utilization of hospital delivery service. In a word, MHC programs (policies) for central and western rural areas have improved the service capacity of primary medical institutions and MHC utilization. Due to these programs and policies, MHC utilization researches have been more diverse with service quality evaluation and service effectiveness evaluation methods being more widely used. Moreover, cooperation between researchers and practitioners has been promoted.

***The third phase (2011—present) How to improve the MHC utilization quality: ***With the social, economic development and implementation of MHC programs and policies, the service capacity of primary medical institutions has been improved. MHC relevant indicator gaps across regions and between urban and rural areas have been narrowed. In 2011, the average MMR, ANC rate and hospital delivery rate were almost the same with the nation’s average level.^19^ Therefore, researchers began to shift their attention to the quantity and quality of MHC utilization. Relevant studies fall into two categories: One is comparative analysis of MHC utilization in reality with the national standards of MHC utilization, that the pregnant women should have their first prenatal visit before 13 weeks of gestation and have at least 5 prenatal visits and at least 3 postnatal visits (within 42 days of delivery), exploring influencing factors from the angle of service demand and supple. Liu X^20^ conducted a face-to-face survey of 112 women from 10 provinces’ 45 counties through a multi-stage random sampling. The differences of main MHC service indicators including ANC hospital delivery, and postpartum visits across regions were analyzed and it indicated that MHC utilization seems to be associated with socio-economic and regional factors.

The other category of research is comparative analysis of MHC utilization, includes horizontal comparison of urban and rural areas in a certain period of time and longitudinal comparison of different periods of time in rural areas. For example, Liu XY^21^ carried out an investigation of pregnant women in urban and rural areas in eastern, central and western areas of China. The differences of MHC utilization between the two groups (urban VS rural areas) were analyzed. The quality problems of MHC utilization have caused the concerns from government, academia about achieving the MDG5. Feng XL’s research (inciting data from Chinese Maternal and Child Health Monitoring System) indicated that although China could achieve the MDG5 as scheduled, still there are large gaps of MHC utilization between regions with uneven economic development.^22^

With the widening of research themes and problems, the research perspectives of central and western China rural areas MHC utilization are becoming more and more diverse, with the research dimension expanding from individual to the service institution. For example, Bright I’s research of central and western rural China MHC utilization indicated that the content of MHC service exerts an on-going influence on the timing of prenatal care initial use and sustainability of prenatal care.^23^ In addition, some researches put MHC utilization in a larger social, economic, and cultural framework, taking women’s family status, decision-making rights, and family planning policy into consideration. For example, Li J’s research of Dian dong county in Yunnan Province (employing fieldwork method) showed that factors influencing MHC utilization were not confined to family income and distance from home to hospitals, but also included women family status and family planning factors.^24^ Although the research themes and target problems of MHC utilization differ in different periods of time, there is no doubt that MHC utilization is always associated closely with the individual, family, community, and service institutions. We summarized the different research perspectives and dimensions under each certain perspective, as shown in Table-II.

**Table-II:** Perspectives and dimensions of MHC research

*Perspectives*	*Dimensions*				
Individual	Population sociological features	Awareness of and attitude towards MHC	Awareness of MHC importance	Pregnancy History	
Family	Economic state	Family members’ awareness	Family status	Decision-making right	Distance
Community	Publicizing	Health education			
Service institution	Service Capacity	Staff’s competency	Service price	Service content	Service Environment
Others	Unplanned pregnancy	Registered permanent residence			

Quantitative studies are large in numbers. Studies of this kind usually focus on one of the three stages of pregnancy (antenatal, intrapartum, and postpartum), using individual or family information as variables, to explore the influencing factors of MHC utilization with statistical indicator description and parametric test. Qualitative studies are usually presented in the form of case studies. Studies of this kind explore the deep-rooted influencing factors of MHC utilization through investigation of the service supply and demand (mainly using interviews with key persons and key groups). Qualitative studies have been paid much attention by researchers since they can improve the validity of the research. For example, Harris A^25^ after carrying out an in-depth interview with both the service supply and demand sides explored the influencing factors of women’s access and utilization of MHC service through analysis of the actual MHC utilization of minority women in Sichuan province. With the introduction of qualitative studies, qualitative data analysis methods, such as theme summarizing method, content analyzing method,^26^ and matrix method, have been further explored. Mixed method aims to explore the in-depth problems and the reasons behind and to discover the characteristics and laws of the subjects’ formation and development.^27^ It is favored by many researchers because it combines the advantages of both quantitative studies and qualitative studies. Tao F^28^ used mixed method to study the postnatal care in two rural counties in Anhui Province, exploring factor that lead to low postpartum visits rate.

***Major achievements and problems concerning MHC utilization: ***Due to the development of social economy, government’s increasing input, implementation of MHC programs, as well as ongoing attention from researchers, considerable achievements have been made: local governments regard MHC as priority of their work and issued related favorable polices^29^; staff competence, facility, and service capacity of primary medical institutions have been greatly improved^30^; pregnant women and their families attach much importance to and employ MHC, taking the initiative to seek MHC service provided by institutions.^31^

Literature analysis found out that although the average level of MHC utilization in central and western rural areas is increasing yearly, the MMR differ greatly across regions due to the fact that primary medical institutions in central and western rural areas fall behind those of cities’ in facility and soft power. For example, the average MMR of an autonomous prefecture in Sichuan Province from 2000 to 2009 is 269.97/100,000, being the highest of China.^32^ The problems in MHC utilization and the severity of the problems differ across regions in central and western rural areas. In general, these problems could be divided into four categories.

The first category are natural, geographical and customs cultural factors which hinder MHC utilization (about 30% of the literatures have mentioned). It is prevalent mainly in remote mountainous areas, poverty-stricken areas and ethnic minority areas with backwards service level. For example, Zen H^33^ carried out a demand survey of MHC in a western county. The survey showed that geographic accessibility and cultural customs are the main factors influencing MHC utilization. The implementation of SHCRW policy from 2008 has made it possible that the subsidize could cover the cost of hospital delivery, which means that pregnant women do not need to worry about the cost. Therefore, in underdeveloped areas, it is objective factors that impede MHC utilization. 

The second category concerns with the contradiction between supply and demand, which means that the MHC service provided by medical institutions cannot meet the demands of pregnant women (about 26% of the literatures have mentioned). It is prevalent in economically and culturally well developed areas. For example, Nong X^34^ carried out a research of MHC utilization in central areas. The research indicates that while pregnant women are satisfied with current MHC service, they expect more comprehensive MHC service. The contradiction is particularly evident in Maternal and child hospital (MCH) of county-level. Statistics show that MHC of county-level, the once main medical service provider, has lost its leadership of township hospitals and village clinics with its service capacity declines gradually.^35^ Both the service volume and patients numbers of the MCH of county are declining since it cannot meet the demands of pregnant women for MHC service.

The third category is concerned with inadequate staff of the primary medical institutions (about 15% of the literatures have mentioned). It is particularly evident in township and village medical institutions. Currently, most township clinics have only one health care worker responsible for MHC administration of all the pregnant women during the whole pregnancy; Most villages have only one village doctor responsible for the health education of all the pregnant women in the village and supposed to provide home service. For towns with a large population, staff shortage problem hinders the providing of MHC service to a certain extent and thus is unfavorable to the pregnant women’s utilization of it. 

The fourth category is concerned with the quantity and quality of MHC utilization (about 10% of the literatures have mentioned). The problem is the utilization of MHC service is not strictly in accordance with MOH standards. For example, Liu Tie analyzed the ANC results of 700 pregnant women in a county of western China and found that the ANC frequency and quality of early pregnancy check rate was poor. The main influencing factors include previous pregnancy, unplanned pregnancy, and whether having joined NRCMS or not.^36^

## DISCUSSION

How to solve the MHC utilization problems in Chinese central and western rural areas? In view of the large differences across regions in the MHC utilization in central and western rural areas, as well as current achievement of MHC programs, we should, first of all, keep focusing on the weakest areas and the most disadvantaged population in terms of economic, natural, geographical, cultural conditions and service capacities to carry out ongoing intervention programs dealing with regional issues to provide funding, resources, health education and other support. It is particularly important to the pregnant women in poor rural areas with weak anti-risk ability. Besides, with the increase of hospital delivery rate, places of maternal death have been shifted to primary health care institutions. Since the cost of rescuing in case of pregnancy and childbirth complications is far beyond the affordability of an ordinary family, a part of fund could be spared from the intervention programs exclusively for covering the cost of rescuing and other emergencies. Thirdly, women’s increasing utilization of MHC service has placed higher demands on the serving capacity of primary health care institutions. However, the inadequate staff number of primary health care institutions is bound to affect the service delivery capabilities and utilization level. Therefore, the government needs to introduce incentive measures to maintain the current staff to provide MHC service which meet both quality and quantity requirement, such as training opportunities or increase salary. Lastly, MHC of county-level which is low in both service capacity and service volume, it is extremely necessary to adopt a more convenient, more effective, more personalized service model, such as going to the countryside regularly, and providing maternal health service at village clinic or at home to ensure that the majority of pregnant women can be guaranteed access to ANC services.

**Fig.2:**
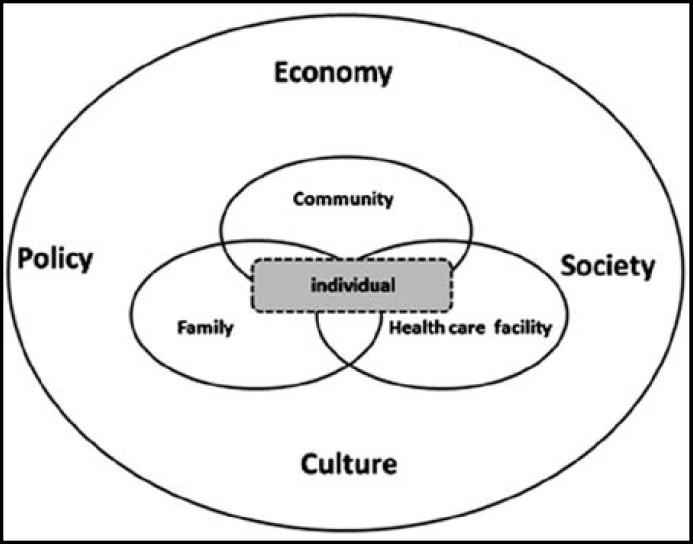
Study perspectives of utilization of MHC

**Fig.3:**
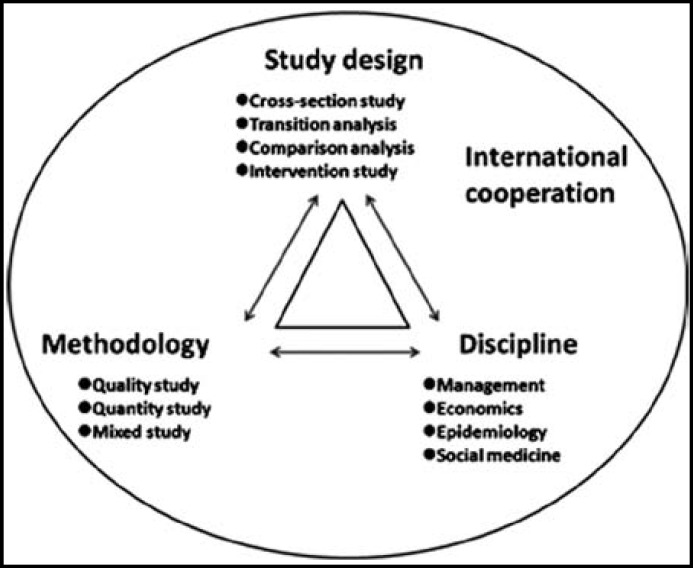
Study design, methodology and discipline of utilization of MHC

How to undertake research of MHC utilization? Having studied the MHC utilization research and MHC practice throughout different phases, it is not difficult to find that the research context shows certain regularity? distinct research themes and research characteristics are presented under different program (policy) orientation or in different periods of time. Research results of the first phase were rarely applied into practical use and also failed to solve the problems of MHC service utilization in Chinese central and western rural areas. But objectively speaking, it is the research of this phase that caused the attention of international community and Chinese government to the importance of MHC in central and western rural areas, promoting the implementation of a series of MHC programs and policies. Due to the implementation of these programs, MHC utilization in central and western rural areas has been improved. Meanwhile, it triggered a large number of studies as impact evaluation of program implementation, thus pushing MHC utilization to a peak. When the implementation has get MMR and other indicators improved, researchers, shifting their attention to the quality of MHC utilization, begin to focus on how to improve the quantity and quality of MHC utilization. In addition, researchers continue to broaden the research perspectives: from the individual to the family, institutions, communities, and other dimensions, and put MHC in a larger economic, social, cultural, and policy-related framework. Fig.2.

Meanwhile, research design, research methodology and different disciplines integrate and connect closely. Also, there is a more evident trend of international cooperation between researchers. Fig.3.

It must be noted that since the MHC research themes, design, content, methods in different period of time or under different program (policy) orientation overlap, therefore the study is not entirely confined to the dimensions the author mentioned. What we strive to present is the trend of research perspective, design, method, and other dimensions. In addition, having compared and analyzed the literature theme and content, we found that the defects of current MHC utilization lie mainly in the following three aspects: Firstly, many researches are repeated and there is a lack of innovation and response to practical problems. Also, evidence-based research is inadequate and many research results have not been accepted and exert no meaningful influence on practice; Secondly, comparison studies between counties are inadequate; thirdly, the research methodology needs to be further discussed. 

To solve practical problems, when carrying out MHC research, firstly, it is necessary to fully understand the characteristic and context of previous research, to avoid repeated research and improve the innovation of future research. Secondly, due to the time lag between research and reality, researchers should enhance cooperation with practitioners and carry out more evidence-based research so as to promote more research findings to be adopted. Thirdly, researchers should carry out more comparative studies between underdeveloped countries (regions) or newly-emerging countries (regions) of similar economic, social and demographic level to obtain more reasonable research design and method. It is also necessary to learn from successful research and build a platform to share cost-effective intervention measures as well as research results. Besides, researchers should avoid any detour in their research and offer effective conference for underdeveloped countries. For example, the medical service provided by MCH of county level not being recognized should be avoided. From the research point of view, widely used research models in the field of health management including structural equation models and multi-level models are rarely used in maternal and child health research. Therefore, for certain research theme and content, researchers could try to employ these models in their studies.

In 2011, the report of the UN MDG5 pointed out that the proportion of women of underdeveloped regions who can get recommended prenatal care has increased from 35% in 1990 to 51% in 2009. Although it has been improved, the percentage is quite small. Therefore, improving MHC utilization is still what underdeveloped countries need to concentrate on. For central and western China and underdeveloped countries, when carrying out relevant research concerning MHC utilization, it is necessary to fully grasp the research context, and make use of the implications of previous research, to improve MHC utilization and thus promote the realization of the MDG5.

## Authors Contribution:

YZ and MXC designed and did statistical analysis and manuscript writing.

ZXW did review and final approval of manuscript.

## Notes

***Source of funding:*** Innovative Research Team in University from Ministry of Education of China [IRT0912], National Natural Science Foundation of China [30872128], “The Dawn” Program of Shanghai Education Commission [08SG08], National ministry of science and technology in the 11th five-year plan funded project of China [2007BAI24B02], Specialized Research Fund for the Doctoral Program of Higher Education of China [20120071110054].
